# Factors Contributing to Lack of Adherence to Antihypertensive Medications Among Patients at Nishtar Hospital in Multan, Pakistan

**DOI:** 10.7759/cureus.69396

**Published:** 2024-09-14

**Authors:** Allahdad Khan, Ayesha Maqbool, Ahmed A Khan, Muhammad Farhan Jamil, Beshair Aziz, Aamir Aziz, Jawad A Khan, Muhammad Hamza Riaz, Wania Naeem, Ahsan Rasheed

**Affiliations:** 1 Department of Medicine, Nishtar Medical University, Multan, PAK; 2 Department of Community Medicine, Nishtar Medical University, Multan, PAK; 3 Department of Family Medicine, Al Barsha Health Center, Dubai, ARE; 4 Department of Internal Medicine, Swansea Bay University Health Board, Swansea, GBR; 5 Department of Medicine, Multan Medical and Dental College, Multan, PAK

**Keywords:** antihypertensive medication, associated factors, high blood pressure, hypertension, medication adherence, nonadherence, pakistan

## Abstract

Background: Hypertension affects 26.7% of Pakistan's population, with only 6% achieving control. This study investigates antihypertensive medication adherence in Multan, focusing on socioeconomic and patient-related factors influencing non-adherence to study the lack of adherence to antihypertensive medications in hypertensive patients and its associated factors at Nishtar Hospital in Multan, Pakistan.

Methods: This cross-sectional study was conducted among hypertensive patients admitted at Nishtar Hospital Multan, Pakistan. Patients under the age of 20 years were excluded from the study. A self-developed questionnaire was used to gather the demographic details of patients. The Hill-Bone Medication Adherence Scale was used via a non-probability convenience sampling technique to deduce the adherence level in patients. Data analysis was done using SPSS (Statistical Package for the Social Sciences) v23 (IBM Corp., Armonk, NY). p-value < 0.05 was considered significant.

Results: Out of 217 respondents, most were female, married, unemployed, and residing in urban areas. Most of them had a higher level of education and a monthly income averaging below 30,000 PKR (Pakistani Rupee). The insight into the hypertensive history showed that most of them had a positive family history and comorbid conditions, and were hypertensive for more than five years. The majority of the patients had a complex regimen prescribed to them with multiple doses throughout the day. The minority were smokers and had medications provided to them for free, through public or government-funded institutions. More importantly, adherence to the antihypertensive therapy was negatively correlated with the age of the patients (p = 0.004, r = -0.195), complexity of regimen (p = 0.041), multiple dosing (p = 0.039), and cost of medication (p = 0.043). All of these relations were statistically significant.

Conclusion: Lack of adherence to antihypertensive medications in hypertensive patients is more common in populations belonging to older age groups, complex regimens, multiple doses, and higher medicine fees.

## Introduction

The World Health Organization (WHO) defines hypertension, or high blood pressure, as a condition where blood pressure readings, when measured on two different occasions, are recorded as 140/90 mmHg or higher, indicating that either the systolic pressure is at least 140 mmHg or the diastolic pressure is at least 90 mmHg [1]. Globally, around 1.28 billion individuals experience high blood pressure, but only 21% have successfully managed to regulate it [1]. Hypertension (HTN) is estimated to affect 26.7% of the population in Pakistan [2], with only 6% having it under control [3]. Its global prevalence is rising swiftly, with the condition approaching epidemic proportions.

Medications designed to manage hypertension effectively reduce blood pressure (BP), protect against organ damage, prevent cardiovascular disease, and decrease mortality rates [4]. Adhering strictly to prescribed antihypertensive therapy can significantly decrease the likelihood of experiencing myocardial infarction (20%-25%), heart failure (more than 50%), and stroke (35%-40%) [5]. Failure to follow prescribed antihypertensive treatments hinders achieving ideal health results. The act of skipping doses of medication during the treatment is called non-adherence. A study conducted in Islamabad, Pakistan's capital city, found that 37.7% of hypertensive patients in primary, secondary, and tertiary healthcare facilities were non-adherent to their treatment plans [5].

Numerous factors contribute to non-adherence to antihypertensive medications. WHO has identified five primary categories for these factors: the socioeconomic status of the patient, aspects of the therapy itself, characteristics of the healthcare system, health-related conditions, and factors specific to the patient [6]. The socioeconomic status is intriguing due to its links with economic, social, and educational factors. These elements collectively impact the consistent use of medication [7]. Moreover, the interaction dynamics between physicians and patients have been proven to be crucial in influencing treatment adherence. Transitioning toward a collaborative and less authoritarian approach is thought to improve overall care quality, patient satisfaction, and adherence to treatment protocols [8]. Furthermore, sufficient social support and interaction among patients and their families have been demonstrated to promote the adoption of beneficial health practices and enhance patients' quality of life [9]. Patients' adherence to treatment is also hindered by the complexity of medication regimens [10] and the number of doses [11], resulting in difficulties in maintaining consistent adherence to treatment.

Addressing the detrimental effects linked to inadequate adherence to antihypertensive medications, which include adverse health outcomes and unnecessary strain on healthcare resources, necessitates a thorough exploration of the underlying causes influencing this behavior. This study aims to examine the adherence levels to antihypertensive medication among hypertensive patients at Nishtar Hospital, Multan. Additionally, it seeks to assess how various socioeconomic factors, patient characteristics, and medical conditions interact to impact non-adherence to antihypertensive treatment.

## Materials and methods

This descriptive cross-sectional study was conducted at Nishtar Hospital, Multan, Pakistan, for a period of eight weeks from June 5, 2024, to July 30, 2024. The sample size was 217, which was calculated by the Epi info tool using a confidence level of 95% and a margin of error of 5% [12]. The study population consisted of previously diagnosed hypertensive patients admitted at the Nishtar Hospital, selected through non-probability convenience sampling. Data was collected after obtaining written informed consent from the patients. Patients under the age of 20 were excluded from the study. A complex regimen was considered to be two or more different antihypertensive drugs used by the patient on a daily basis. Multiple doses were considered as taking two or more doses of the same antihypertensive drug in a day.

A questionnaire was developed to gather demographic details of the patients. The Hill-Bone Medication Adherence Scale (HB-MAS) was used to deduce the level of adherence in patients [13]. A pilot study was conducted on 20 patients to test the feasibility of our research methods and procedure. The responses to the pilot study were excluded from the final results but were used to improve the questionnaire depending on the feedback from the patients. The responses were recorded in a spreadsheet on Microsoft Excel (Microsoft Corp., Redmond, Washington), which underwent further biostatistical analysis on SPSS (Statistical Package for the Social Sciences) v23 software (IBM Corp., Armonk, NY) . Pearson's correlation was used to find out the relationship between the age of the patients and their adherence scores (indicated by their HB-MAS score). Multiple linear regression analysis was performed to determine the effect of the complexity of regimens, the number of doses, and the cost of medication (information obtained in the questionnaire) on the adherence scores (calculated through the HB-MAS scale) of patients. The p-value < 0.05 was considered statistically significant.

## Results

A total of 217 respondents participated in the study. Of the total respondents, the majority of the participants were aged 40-59 years (92 participants, 42.4%), female (123 participants, 56.7%), and urban residents (137 participants, 63.1%). According to their responses, 139 (64.1%) were unemployed, 173 (79.7%) were married, 92 (42.4%) had monthly incomes averaging below 30,000 PKR (Pakistani Rupee), and 68 (31.3%) had a higher level of education, while the remaining had only completed primary, middle, and secondary levels of education (Table 1).

**Table 1:** Demographic information of the patients The data are expressed as frequencies (N) and percentages (%).

Measure	Item	Frequency (n = 217)	Percentage
Age	20-39 years	45	20.7%
	40-59 years	92	42.4%
	Above 60 years	80	36.9%
Gender	Male	94	43.3%
	Female	123	56.7%
Occupational status	Employed	78	35.9%
	Unemployed	139	64.1%
Marital status	Married	173	79.7%
	Unmarried	44	20.3%
Education level	Grades 1-5	74	34.1%
	Middle school (Grades 6-8)	25	11.5%
	Matriculation (Grades 9-10)	35	16.1%
	Intermediate (Grades 11-12)	15	6.9%
	Higher	68	31.3%
Residential area	Rural	80	36.9%
	Urban	137	63.1%
Average income	<30k	92	42.4%
	30k-70k	73	33.6%
	>70k	52	24.0%

The gathered data was evaluated for the frequencies of participants’ responses to get an insight into the hypertensive history of the sample. As illustrated in Table 2, 135 participants (62.2%) had a positive family history of hypertension, 123 (56.7%) were positive for comorbid conditions, and 86 (39.6%) had been hypertensive for more than five years. The hypertensives were taking medications to control hypertension, of which 93 participants (42.9%) had a complex drug regimen comprising multiple drugs, with 85 (39.2%) of them taking multiple doses during the day. Only 44 participants (20.3%) had antihypertensive medications provided to them for free, through public or government-funded institutions. Interestingly, only a minority of 49 respondents (22.6%) of the hypertensive population were smokers.

**Table 2:** History of disease under study (hypertension) The data are expressed as frequencies (N) and percentages (%).

Questions	Answers	Frequency (n = 217)	Percentage
Family history of hypertension	Yes	135	62.2%
	No	82	37.8%
Comorbid conditions	Yes	123	56.7%
	No	94	43.3%
Time since diagnosis of hypertension	<1 year	46	21.2%
	Between 1 and 5 years	83	38.2%
	5 years	2	0.9%
	>5 years	86	39.6%
Complex regimen	Yes	93	42.9%
	No	124	57.1%
Multiple doses	Yes	85	39.2%
	No	132	60.8%
Cost of medicine	Free	44	20.3%
	Pay yourself	173	79.7%
Smoker	Yes	49	22.6%
	No	168	77.4%

It was deemed important to understand whether complex regimens, multiple doses, advancing age, or bearing the cost of medications contributed significantly to patients' non-compliance. Pearson’s correlation (two-tailed) was used with a confidence interval of 95% to find out the correlation between age and the adherence of patients to antihypertensive therapy (represented by HB-MAS scores). The scores of participants' antihypertensive treatment compliance (HB-MAS scores) were weakly negatively correlated to the age of participants (p = 0.004, r = -0.195), suggesting a greater degree of non-adherence in the hypertensive populations belonging to older age groups. This correlation was statistically significant (p < 0.05).

A multiple regression analysis was conducted (Table 3) to examine the relationship between patient adherence to antihypertensive therapy, as measured by HB-MAS scores, and the factors hypothesized to influence treatment adherence, such as complexity of regimen, number of doses, and the cost of medication. This analysis allowed us to determine the individual impact of each factor on adherence while accounting for the influence of the other factors. As shown in Table 3, all three variables (complex regimen, multiples doses, and medicine fees) show a negative relation with patient’s adherence to antihypertensive therapy as measured by the HB-MAS scores. All of these relations were statistically significant (p = 0.041, p = 0.039 and p = 0.043, respectively). The data shows how relatively simple treatment regimens, fewer doses, and reduced medicine fees were associated with better adherence in patients.

**Table 3:** Relationship of complexity of regimens, number of doses, and cost of medication with HB-MAS score Data are expressed as unstandardized coefficients (b, standard error), standardized coefficients (beta), t-values (t), and significance (p-value). p-value < 0.05 was considered statistically significant. HB-MAS: Hill-Bone Medication Adherence Scale.

	Unstandardized coefficients		Standardized coefficients (Beta)	t	Significance (p-value)
	B	Std. error			
Complex regimen	-0.638	0.311	-0.141	-2.054	0.041
Multiple doses	-0.728	0.350	-0.160	-2.078	0.039
Medicine fee	-0.719	0.354	-0.150	-2.032	0.043

## Discussion

This study on the factors contributing to non-adherence to antihypertensive medication is one of the few conducted in the South Punjab region of Pakistan. Non-adherence of patients to their prescribed antihypertensive regimens has significant negative implications for their treatment efficacy and overall patient health. Several factors were identified as contributing to this non-adherence, including advancing age, number of doses, complexity of regimens, and the cost of medication.

A significant portion of our participants, 172 (79.3%), were aged 40 years and above, with 80 (36.9%) older than 60 years. Our results indicate a clear trend of decreasing adherence with increasing age (p = 0.004, r = -0.195). This is likely due to issues such as cognitive deficits and the burden of managing concurrent treatments [10] for other conditions that develop with advancing age like diabetes mellitus. As age increases, the prevalence of these challenges also increases, leading to missed doses or complete neglect of medication.

Complex medication regimens (having two or more antihypertensive drugs) were another significant factor, with 93 (42.9%) of patients on such regimens. Polypharmacy (use of five or more drugs), the development and availability of several drugs on the market, and disease pattern shifts are factors that have contributed to the emergence of complex drug therapies [14].

The complexity of regimens was found to be associated with lower adherence to medications among patients (p = 0.041). This can be due to the increased time and effort required to manage and remember multiple medications, leading to treatment fatigue. Moreover, patients with these diseases do not always have symptoms that help to remind them of the need to use the drugs appropriately [14].

The study also found that 85 (39.2%) patients were taking multiple doses of the same medication daily. An increasing number of doses was associated with lower adherence among patients (p = 0.039), possibly due to the psychological burden and financial strain. Additionally, adverse effects experienced during therapy are a major reason for non-adherence [15]. Increasing the number of doses increases these adverse effects. Consequently, patients often modify their treatment plans to suit their convenience, which may not align with the prescribed regimen, thereby compromising their treatment.

The cost of medication is another substantial barrier to adherence. Patients who had to purchase their medication, as was the case for 173 (79.7%) of our participants, are less likely to adhere to their treatment plans, as indicated by this study (p = 0.043). High medication costs often lead patients to alter their regimens based on financial constraints rather than medical advice [16]. A systematic review conducted in 2023 also demonstrated how low socioeconomic level is the leading barrier to HTN management in Pakistan [3].

This study is significant as it studies the demographic characteristics and hypertensive history as well as provides a comprehensive analysis of the factors contributing to non-adherence to antihypertensive medication in the South Punjab region of Pakistan, a topic that has been underexplored in this geographical context. The findings emphasize the need for simplified treatment regimens, affordable medication, and targeted interventions for older adults and economically disadvantaged populations. This study not only contributes valuable insights for healthcare providers to improve patient compliance but also informs policymakers about the critical areas requiring attention to enhance public health outcomes in the region.

Limitations

Despite its contributions, this study has several limitations that should be acknowledged. First, the research was confined to participants from a single medical facility in Multan, which may limit the generalizability of the findings to other parts of South Punjab or Pakistan. Additionally, the study relied on self-reported data, which can be subject to recall bias and social desirability bias, particularly when assessing medication adherence and health behaviors. Moreover, the cross-sectional design of the study limits our ability to establish causality between the identified factors and medication adherence. Lastly, a lack of proper knowledge about the disease and education among the patients was also an obstacle in conducting this study.

Moving forward, future research endeavors should aim to address these limitations and expand upon the findings of this study. Conducting multicenter studies across diverse regions of South Punjab would help validate the current findings and provide a more comprehensive understanding of regional variations in medication adherence. Furthermore, incorporating digital health technologies and telemedicine interventions could offer innovative solutions to support patients in managing their medication regimens effectively, particularly in rural areas with limited access to healthcare resources. These future endeavors hold promise in advancing our knowledge and improving health outcomes for hypertensive patients in the region.

## Conclusions

In conclusion, this study focused on hypertensive patients’ lack of adherence to antihypertensive medications and the associated factors at Nishtar Hospital in Multan, Pakistan. Most participants were female, unemployed, married, and urban residents with monthly incomes averaging below 30,000 PKR. The insight into the hypertensive history of the sample showed that most had a positive family history of hypertension; a majority were positive for comorbid conditions, and a significant portion of respondents had been hypertensive for more than five years. Hypertensive populations belonging to older age groups, complex regimens, multiple doses, and higher medicine fees showed a statistically significant negative relation with adherence to antihypertensive therapy.

## Appendices

**Table 4:** The Hill-Bone Medication Adherence Scale (HB-MAS) used in the questionnaire

No.	Item	Response
1	How often do you forget to take your high blood pressure medicine?	1. All of the time
		2. Most of the time
		3. Some of the time
		4. None of the time
2	How often do you decide NOT to take your high blood pressure medicine?	1. All of the time
		2. Most of the time
		3. Some of the time
		4. None of the time
3	How often do you forget to get prescriptions filled?	1. All of the time
		2. Most of the time
		3. Some of the time
		4. None of the time
4	How often do you run out of high blood pressure pills?	1. All of the time
		2. Most of the time
		3. Some of the time
		4. None of the time
5	How often do you skip your high blood pressure medicine before you go to the doctor?	1. All of the time
		2. Most of the time
		3. Some of the time
		4. None of the time
6	How often do you miss taking your high blood pressure pills when you feel better?	1. All of the time
		2. Most of the time
		3. Some of the time
		4. None of the time
7	How often do you miss taking your high blood pressure pills when you feel sick?	1. All of the time
		2. Most of the time
		3. Some of the time
		4. None of the time
8	How often do you take someone else's high blood pressure pills?	1. All of the time
		2. Most of the time
		3. Some of the time
		4. None of the time
9	How often do you miss taking your high blood pressure pills when you are careless?	1. All of the time
		2. Most of the time
		3. Some of the time
		4. None of the time
